# A Path Toward Precision Medicine for Neuroinflammatory Mechanisms in Alzheimer's Disease

**DOI:** 10.3389/fimmu.2020.00456

**Published:** 2020-03-31

**Authors:** Harald Hampel, Filippo Caraci, A. Claudio Cuello, Giuseppe Caruso, Robert Nisticò, Massimo Corbo, Filippo Baldacci, Nicola Toschi, Francesco Garaci, Patrizia A. Chiesa, Steven R. Verdooner, Leyla Akman-Anderson, Félix Hernández, Jesús Ávila, Enzo Emanuele, Pedro L. Valenzuela, Alejandro Lucía, Mark Watling, Bruno P. Imbimbo, Andrea Vergallo, Simone Lista

**Affiliations:** ^1^Sorbonne University, GRC no. 21, Alzheimer Precision Medicine (APM), AP-HP, Pitié-Salpêtrière Hospital, Boulevard de l'hôpital, Paris, France; ^2^Department of Drug Sciences, University of Catania, Catania, Italy; ^3^Oasi Research Institute—IRCCS, Troina, Italy; ^4^Department of Neurology and Neurosurgery, McGill University, Montreal, QC, Canada; ^5^Department of Anatomy and Cell Biology, McGill University, Montreal, QC, Canada; ^6^Department of Pharmacology and Therapeutics, McGill University, Montreal, QC, Canada; ^7^Department of Pharmacology, University of Oxford, Oxford, United Kingdom; ^8^Laboratory of Neuropharmacology, EBRI Rita Levi-Montalcini Foundation, Rome, Italy; ^9^School of Pharmacy, Department of Biology, University of Rome Tor Vergata, Rome, Italy; ^10^Department of Neurorehabilitation Sciences, Casa Cura Policlinico, Milan, Italy; ^11^Brain & Spine Institute (ICM), INSERM U 1127, CNRS UMR 7225, Boulevard de l'hôpital, Paris, France; ^12^Institute of Memory and Alzheimer's Disease (IM2A), Department of Neurology, Pitié-Salpêtrière Hospital, AP-HP, Paris, France; ^13^Department of Clinical and Experimental Medicine, University of Pisa, Pisa, Italy; ^14^Department of Biomedicine and Prevention, University of Rome “Tor Vergata”, Rome, Italy; ^15^Department of Radiology, “Athinoula A. Martinos” Center for Biomedical Imaging, Boston, MA, United States; ^16^Harvard Medical School, Boston, MA, United States; ^17^Casa di Cura “San Raffaele Cassino”, Cassino, Italy; ^18^NeuroVision Imaging, Inc., Sacramento, CA, United States; ^19^Centro de Biología Molecular Severo Ochoa (CSIC-UAM), Madrid, Spain; ^20^Network Center for Biomedical Research in Neurodegenerative Diseases (CIBERNED), Madrid, Spain; ^21^2E Science, Robbio, Italy; ^22^Systems Biology Department, University of Alcalá, Madrid, Spain; ^23^Faculty of Sport Sciences, Universidad Europea de Madrid, Madrid, Spain; ^24^Research Institute of the Hospital 12 de Octubre (“imas”), Madrid, Spain; ^25^Centro de Investigación Biomédica en Red Fragilidad y Envejecimiento Saludable (CIBERFES), Madrid, Spain; ^26^TranScrip Partners, Reading, United Kingdom; ^27^Research & Development Department, Chiesi Farmaceutici, Parma, Italy

**Keywords:** Alzheimer's disease, neuroinflammation, microglia, neuroinflammatory pathways, biomarkers, anti-inflammatory therapy, systems biology, precision medicine

## Abstract

Neuroinflammation commences decades before Alzheimer's disease (AD) clinical onset and represents one of the earliest pathomechanistic alterations throughout the AD continuum. Large-scale genome-wide association studies point out several genetic variants—*TREM2, CD33, PILRA, CR1, MS4A, CLU, ABCA7, EPHA1*, and *HLA-DRB5-HLA-DRB1*—potentially linked to neuroinflammation. Most of these genes are involved in proinflammatory intracellular signaling, cytokines/interleukins/cell turnover, synaptic activity, lipid metabolism, and vesicle trafficking. Proteomic studies indicate that a plethora of interconnected aberrant molecular pathways, set off and perpetuated by TNF-α, TGF-β, IL-1β, and the receptor protein TREM2, are involved in neuroinflammation. Microglia and astrocytes are key cellular drivers and regulators of neuroinflammation. Under physiological conditions, they are important for neurotransmission and synaptic homeostasis. In AD, there is a turning point throughout its pathophysiological evolution where glial cells sustain an overexpressed inflammatory response that synergizes with amyloid-β and tau accumulation, and drives synaptotoxicity and neurodegeneration in a self-reinforcing manner. Despite a strong therapeutic rationale, previous clinical trials investigating compounds with anti-inflammatory properties, including non-steroidal anti-inflammatory drugs (NSAIDs), did not achieve primary efficacy endpoints. It is conceivable that study design issues, including the lack of diagnostic accuracy and biomarkers for target population identification and proof of mechanism, may partially explain the negative outcomes. However, a recent meta-analysis indicates a potential biological effect of NSAIDs. In this regard, candidate fluid biomarkers of neuroinflammation are under analytical/clinical validation, i.e., TREM2, IL-1β, MCP-1, IL-6, TNF-α receptor complexes, TGF-β, and YKL-40. PET radio-ligands are investigated to accomplish *in vivo* and longitudinal regional exploration of neuroinflammation. Biomarkers tracking different molecular pathways (body fluid matrixes) along with brain neuroinflammatory endophenotypes (neuroimaging markers), can untangle temporal–spatial dynamics between neuroinflammation and other AD pathophysiological mechanisms. Robust biomarker–drug codevelopment pipelines are expected to enrich large-scale clinical trials testing new-generation compounds active, directly or indirectly, on neuroinflammatory targets and displaying putative disease-modifying effects: novel NSAIDs, AL002 (anti-TREM2 antibody), anti-Aβ protofibrils (BAN2401), and AL003 (anti-CD33 antibody). As a next step, taking advantage of breakthrough and multimodal techniques coupled with a systems biology approach is the path to pursue for developing individualized therapeutic strategies targeting neuroinflammation under the framework of precision medicine.

## Introduction

Alzheimer's disease (AD) is the most commoncause of neurodegenerative dementia. According to current estimates, 17% of people aged 75–84 years in the United States have AD, and the disease costs the country US$236 billion per year. The prevalence is projected to triple by 2050 to >15 million, with annual costs of >$700 billion (1). There is an urgent need for developing pharmacological treatments with a disease-modifying effect to halt the disease at its earliest preclinical stage where brain and cognitive functions can still be preserved (2, 3). Indeed, drugs currently available on the pharmaceutical market (i.e., acetylcholinesterase inhibitors and non-competitive *N*-methyl-d-aspartate antagonists) have been approved for a symptomatic effect only and for the dementia stage of AD (4).

The acknowledged pathophysiological hallmarks—(I) extracellular deposition of amyloid beta (Aβ), (II) intracellular aggregates of tau proteins, ultimately called neurofibrillary tangles (NFT), and (III) neurodegeneration—have been integrated in research diagnostic criteria (5–8).

The hypothesis-free biomarker-guided “A/T/N” classification scheme was introduced to categorize subjects based on core AD hallmarks (9). The A/T/N scheme is anticipated to provide consistent recruitment of individuals and target engagement among various different sites in AD clinical trials. Even though the A/T/N classification scheme provides crucial pathophysiological insights, it offers a partial depiction of the *spectrum* of pathomechanistic modifications of AD (10, 11).

The increasing animal and in-human evidence for the upstream role that neuroinflammation may play in AD has posed several conceptual therapeutic concerns and open up new avenues for preventing AD cognitive decline.

The pathophysiological mechanisms of multifactorial and polygenic AD are not limited to the neuronal tissue; they are related to cerebral immunological responses (12). Indeed, brains of patients with AD and other neurodegenerative diseases (ND) show chronic inflammation (13). Neuroinflammation is as an innate immunological response of the nervous system that comprises microglia, astrocytes, cytokines, and chemokines, which play a central role in an early phase of AD pathogenesis (12, 14). The key contribution of inflammation in the AD pathophysiology has been hypothesized more than 20 years ago (12, 15–17). Recent studies demonstrate that this early disease-aggravating central nervous system (CNS) inflammation starts decades before the appearance of severe cognitive decay or AD (18–20). Along this line, different longitudinal studies show that inflammation and microglial activation occur years before AD onset (21–23). Furthermore, there is a strong link between neuroinflammation and amyloid and tau accumulation in the human brain (23–26).

The acknowledged cell mediators of inflammatory mechanisms in AD are microglia and astrocytes (12). In general, these cells play a substantial role in neural transmission and synapse remodeling, as they facilitate the removal of non-essential synapses by eradicating inadequate connections (27, 28). Thus, the efficiency of neuronal transmission is increased.

## Neuroinflammation and Cell Mediators of Inflammatory Mechanisms in Alzheimer'S Disease

### The Role of Microglia and Astrocytes in Alzheimer's Disease Synaptic Dysfunction

Synapses exhibit a quad-partite arrangement that consists of an axon terminal, a dendritic spine put in direct communication with a microglial and an astrocytic process (29). Astrocytes and microglia—the brain-resident macrophages—play a key role in neural circuit development and synaptic homeodynamics during adulthood. Astrocytes are essential for supporting synaptogenesis (axonal and dendritic spines sprouting) and regulating synaptic robustness (30–32). Astrocytes also contribute to the spatiotemporal integration of several synaptic signals and regulate the synaptic transmission (33, 34). Microglial cells play a key role in the immune surveillance of the presynaptic microenvironment and also for the synaptic remodeling toward axonal and dendritic terminals pruning by reshaping proteolytic and phagocytic processes. Microglial cells are able to recruit astroglia, or they can be recruited by the latter (30–32, 35). They are thought to drive the well-known age-related regional synaptic vulnerability, as recently reported (36). Indeed, an age-related ultrastructural and functional shift of microglia cells is associated with increased synaptic susceptibility and neurodegeneration (35).

Therefore, astrocytes and microglia express physiological properties essential for synaptic transmission, the accurate modulation of neural and synaptic plasticity, and both synaptic adaptation and homeostasis (30–32).

In summary, it is well-established that microglia and astrocytes take part in aberrant molecular pathways that, ultimately, reflect AD pathomechanistic alterations, i.e., brain proteinopathies, synaptic failure, loss of brain plasticity, neuroinflammation, axonal damage, and neurodegeneration (37–41).

### The Role of Microglia

Microglial cells, arising from the mesodermal (myeloid) lineage (42), are the main category of macrophages in the CNS parenchyma. They express a large assortment of receptors that recognize exogenous or endogenous CNS insults and initiate an immune response. Besides their typical immune cell role, microglial cells protect the brain by stimulating phagocytic clearance and providing trophic sustenance to preserve cerebral homeostasis and support tissue repair. When circumstances related to loss of homeostasis or tissue alterations occur, then many dynamic microglial mechanisms are triggered, leading to the “activated state” of microglia (43). These encompass cellular morphology modifications, changes in the secretory profile of molecular mediators, and increased proliferative responses (44). A persistent homeodynamic imbalance, such as brain accumulation of Aβ, can trigger a step further in activation, referred to as “priming” (37). Priming of microglia is directed by alterations in their microenvironment and the release of molecules guiding their proliferation. Priming makes microglia inclined to secondary inflammatory stimulating factors, which can then elicit amplified inflammatory reactions (37).

Activated microglia is a typical pathophysiological feature of AD and other ND (12, 43, 45). Two main types of microglia cells are present in the brain, “resting” (or “quiescent”) and “active” microglia. In particular, there is evidence for the high degree of heterogeneity of microglial activation in the CNS, which can be categorized into two opposite activation phenotypes: M1 and M2 (43, 46, 47). According to the phenotype activated, microglia can generate either cytotoxic or neuroprotective effects (46). The M1 or “proinflammatory” phenotype (classically activated) displays proinflammatory cytokines and nitric oxide. It decreases the release of neurotrophic factors, thus exacerbating inflammation and cytotoxicity (43). In contrast, the M2 or “anti-inflammatory” phenotype (alternatively activated) displays anti-inflammatory cytokines, increased expression of neurotrophic factors, and several other signals involved in downregulation, protection, or repair processes in response to inflammation (43). Preliminary evidence from experimental studies suggests that the phenotypic transformation of the activated M1/M2 functional states (“phenotypic switching”) (48, 49) can be determined by both the stage and the severity of the disease. In preclinical models, M1 microglia seems to prevail at the injury site, at the end stage of disease, and once inflammation resolution and repair processes of M2 microglia are diminished (46).

In light of the increasing evidence that the modality by which microglia is activated is a continuum between proinflammatory (M1) and anti-inflammatory (M2) phenotypes, the M1/M2 “dichotomy” (or “polarization” scheme) is still disputed. Actually, it seems possible that the global process of microglia activation represents a much larger heterogeneous *spectrum* of very dissimilar responses (43).

Experimental models of AD demonstrate that microglia cluster around plaques, likely via chemotactic mechanisms, and may contribute both in Aβ (39, 44) clearance and in limiting the growth and further accumulation plaques (39, 44). Moreover, the dysregulation of microglia activity, including dystrophic microglia, may be either a trigger, or a worsening factor, or both, of the seeding of aberrant protein aggregates in the brain (39, 44).

**Figure 1:**
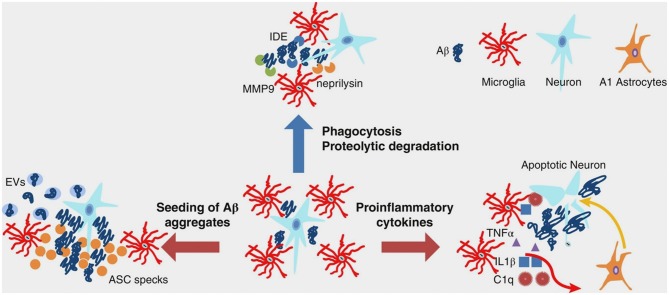
Multifaceted functions of microglia during Aβ pathology. In healthy brain and early stages of AD, microglia clear small aggregates of Aβ peptides by phagocytosis and by secreting proteolytic enzymes, such as IDE, neprilysin, and MMP9. During advanced AD, microglia exacerbate AD pathology by releasing proinflammatory cytokines that induce neuronal cell death as well as A1 astrocytes, which, in turn, affect neuronal survival. Moreover, during advanced AD, microglia-derived ASC specks and EVs promote seeding of Aβ aggregates. Aβ, amyloid beta; AD, Alzheimer's disease; ASC, apoptosis-associated speck-like protein containing a CARD; C1q, complement component 1q; EVs, extracellular vesicles; IDE, insulin degrading enzyme; IL-1β, interleukin-1 beta; MMP-9, metalloprotease-9; TNF-α, tumor necrosis factor-alpha. From Wang and Colonna (45). Copyright© 2019, Society for Leukocyte Biology. Reprinted with permission from Wiley.

In AD, during inflammation, there is a transition from the resting to the active functional state of microglia that, at a general level, might be the consequence of stress or depressive-like behavior (50). At a molecular level, inflammation is promoted by the presence of Aβ aggregates, including oligomers and fibrils (51–54). Indeed, microglia can bind to soluble Aβ oligomers and insoluble Aβ fibrils through cell surface receptors, including the class A1 scavenger receptor (SCARA1), cell surface cluster of differentiation (CD) markers (CD36, CD14, and CD47), the α6β1 integrin, and the Toll-like receptors (TLRs) (55–58). A key point within the scientific debate is represented by a recent evidence indicating that microglia displays either beneficial or harmful effects throughout the beginning and advancement of AD (45). This is strictly related to the nature of the major activities: (I) clearance of Aβ or (II) release of proinflammatory mediators. In early AD pathogenesis, Aβ oligomers and fibrils gather in the extracellular space and elicit a pathological cascade resulting in neuronal apoptosis and depletion. Microglia eliminate Aβ peptides and dying/dead cells through phagocytosis (59, 60). Besides clearance of Aβ oligomers and fibrils, microglia surrounds plaques and fibrils likely creating a physical barrier that can prevent their spreading and toxicity (61). Aβ clearance is also stimulated by the release of numerous proteases participating in Aβ degradation (62). In spite of the advantageous actions of early activation of microglia cells, their chronic activation by Aβ is detrimental and induces protracted inflammation and disproportionate Aβ deposition, thus rushing neurodegeneration (Figure 1). During AD pathogenesis, the production and release of proinflammatory cytokines and other detrimental components are intensified. In addition, the typical phagocytic action of microglia is decreased. Moreover, the microglial-dependent release of apoptosis-associated speck-like protein containing a caspase recruitment domain (ASC) modulates the diffusion of the pathology within and between cerebral areas (63). Extracellular vesicles—constituted by microvesicles and exosomes and released by reactive microglia—play a role in AD pathogenesis (64) (Figure 1). Finally, microglial cells are able to regulate AD pathogenesis via active interaction with neurons, astrocytes, and oligodendrocytes. Indeed, activated microglial cells induce altered astrocytes via proinflammatory cytokines (Figure 1). These astrocytes can rush and aggravate neuronal and oligodendrocytes death (65).

**Figure 2:**
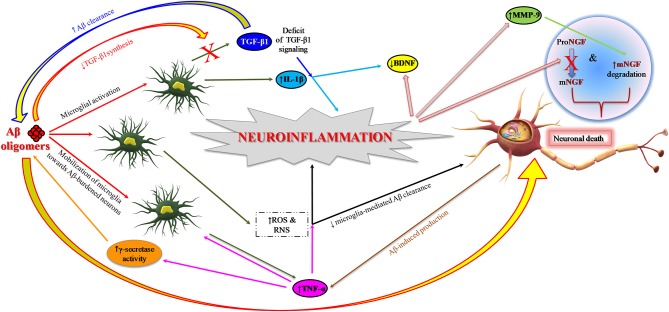
Role of neuroinflammation in AD pathogenesis: impairment of neurotrophin signaling. Aβ_1−42_ oligomers promote neuroinflammation and neuronal death in AD brain by eliciting the release of proinflammatory cytokines (IL-1β and TNF-α) from microglia and also interfering with the synthesis of anti-inflammatory cytokines such as TGF-β1. TNF-α inhibits microglia phagocytosis of Aβ and stimulates γ-secretase activity, thus facilitating Aβ accumulation and microglia-mediated neuroinflammation. Proinflammatory microglial activities promote neuronal death also through the formation of ROS and RNS. Neuroinflammatory phenomena can finally contribute to the pathogenesis of AD by impairing neurotrophin signaling function: (I) reducing the synthesis of BDNF and TGF-β1 and (II) causing an impairment of NGF metabolic pathway characterized by a reduced conversion of proNGF to biologically active mNGF and by an increased degradation of mNGF promoted by MMP-9. Aβ, amyloid beta; Aβ_1−42_, 42-amino acid-long amyloid beta peptide; BDNF, brain-derived neurotrophic factor; IL-1β, interleukin-1 beta; MMP-9, metalloprotease-9; NGF, nerve growth factor; mNGF, mature nerve growth factor; proNGF, precursor of the nerve growth factor; RNS, reactive nitrogen species; ROS, reactive oxygen species; TGF-β, transforming growth factor-beta; TNF-α, tumor necrosis factor-alpha.

The still open question is to understand the specific contributions of neuronal and glial cells in the early phase of inflammation in preclinical AD. Aβ_1−42_ oligomers have a major role in synaptic depletion and gradual cognitive deterioration (66, 67). They induce neuroinflammation and neurodegeneration by stimulating the microglia to produce and release proinflammatory cytokines (14, 68) and also by interfering with the synthesis of anti-inflammatory cytokines, for instance the transforming growth factor-beta 1 (TGF-β1) (Figure 2) (69–71). This early proinflammatory process is characterized by neuronal and microglia-derived cytokines and chemokines as well as by mobilization of microglia toward Aβ-burdened neurons (Figure 2) (19, 72). In addition to Aβ, extracellular non-phosphorylated tau, rather than hyperphosphorylated tau (p-tau), activates the p38 mitogen-activated protein kinase (MAPK) pathway, eliciting a proinflammatory reaction (73).

### The Role of Astrocytes

Astrocytes, differently from microglia and similarly to neurons and oligodendrocytes, arise from the neuroectoderm (74). These cells promotes synaptogenesis (axonal and dendritic spines sprouting), regulates the synaptic strength, take part in the spatial–temporal integration of multiple synaptic processes, and modulate the neurotransmission. Hence, astrocytes execute a variety of physiological activities, in both developing and adult brain, that are essential for synaptic plasticity and a solid and organized cognitive activity (74). Of note, astrocytes modulate Ca^2+^-dependent signaling pathways that are crucial for hippocampal synaptic function and plasticity (75, 76). Indeed, depending on the fluctuations of intracellular Ca^2+^ concentrations, they release gliotransmitters, such as glutamate, D-serine, and ATP, which have feedback actions on neurons (77). Moreover, each astrocyte wraps several neurons, thus interacting with hundreds of neuronal dendrites (78) and connecting with up to two million synapses in the human cortex (79). This kind of interconnectedness indicates that each astrocyte creates a hub to facilitate the integration of the information (74). Moreover, remodeling of astrocytes promotes neuroprotection and recovery of injured neural tissue (80, 81). Along with microglia activation, hypertrophic reactive astrocytes gather around Aβ plaques as reported in human postmortem studies (82) as well as in animal models (83). Like microglia, astrocytes are also activated by tissue injury, infection, and inflammation (84). In AD, after exposure to Aβ, astrocytes release various proinflammatory molecules, such as cytokines, interleukins (ILs), complement components (85–87), nitric oxide, and other cytotoxic compounds, ultimately amplifying the neuroinflammatory response.

Human neuropathological studies conducted on AD brains report the presence of cytoplasmic inclusions of non-fibrillar Aβ in astrocytes, supposed to reflect a phagocytic engulfment from extracellular Aβ deposits (86). In addition, rodent models of AD indicate the ability of astrocytes to uptake and clear Aβ in subjects bearing cerebral fibrillar aggregates and diffuse plaques (16, 17, 33, 86). Conversely, the shutdown of astrocyte-mediated homeodynamics is associated with increased Aβ plaque burden and synaptic terminals dystrophy (68). This enhanced phagocytic activity may represent a compensatory mechanism to incipient increased Aβ accumulation to neutralize its induced toxicity.

## Genes Modulating Neuroinflammation in Alzheimer's Disease

Genome-wide association studies (GWAS) allowed the detection of more than 40 susceptibility gene variants associated with a bigger risk of developing late-onset AD (88). These results include genes associated with immune reaction (in particular, *ABCA7, CD33, CLU, CR1, EPHA1, HLA-DRB5-HLA-DRB1*, and *MS4A*). The relevance of neuroinflammation is further sustained by recent large-scale GWAS showing that the risk of developing late-onset AD is substantially more elevated in individuals with rare variants of microglial immunoreceptors: *TREM2*, encoding the triggering receptor expressed on myeloid cells 2 protein (89); *CD33* (transmembrane receptor CD33), expressed on cells of myeloid lineage (90, 91); and *PILRA* (paired immunoglobulin-like type 2 receptor alpha) (92).

The receptor protein TREM2 enhances the rate of phagocytosis in microglia and macrophages, modulates inflammatory signaling, and controls myeloid cell number, proliferation, and survival (89). Recent studies show that triggering TREM2 receptor in microglial cells is closely associated with the pathogenesis of AD (93). TREM2 modulates microglial functions (e.g., stimulates the production of inflammatory cytokines) in response to Aβ plaques and tau tangles (94, 95). TREM2 absence enhances amyloid pathology, during early AD; however, this is exacerbated at later stages due to the loss of phagocytic Aβ clearance (94). *TREM2* variants cause AD by decreasing the Aβ phagocytic ability of microglia and through the dysregulation of the proinflammatory response of these immune cells (96). Interestingly, the analysis of the existing single-cell transcriptome datasets for human neurons highlights the association of microglia with late-onset AD (97). In addition, the study of regulatory networks of genes showing differential expression in AD brains indicates that immune- and microglia-specific gene modules primarily contribute to AD pathophysiology (98). Finally, Tanzi and colleagues, after exploring the potential role of the cross-talk between CD33 and TREM2 in both neuroinflammation and the cause of AD, propose that TREM2 is working downstream of CD33 to modulate the neuroinflammatory process (99).

## Role of Neuroinflammation in Adult Neurogenesis and Alzheimer's Disease

Besides the above-mentioned role of Aβ and tau in triggering neuroinflammation, it is assumed that the presence of extracellular tau plays a role in the transition from resting to active microglia. In the resting microglia, the protein fractalkine (CX3CL1), secreted by healthy neurons, binds to the cell receptor (CX3CR1) present in the microglia allowing the maintenance of microglia in the resting state. Tau pathology is shown to be associated with neuroinflammatory processes. On the other hand, microglia could be involved in tau propagation in tauopathies. In this scenario, microglial CX3CR1 acts like a receptor for extracellular tau, since the absence of CX3CR1 impairs the internalization of tau microglia (100). Thus, extracellular tau can compete with CX3CL1 for a common receptor. Microglia cells lacking CX3CR1 are deficient in neuronal CX3CL1 signaling and are not in the resting state. As a result, these active microglial cells could secrete some compounds, such as cytokines, potentially affecting neuronal functions like adult neurogenesis. The absence of the microglial CX3CR1 impairs the synaptic integration of adult born hippocampal granule neurons (101). Mice lacking CX3CR1 show modifications in both microglia and neurons of some cerebral areas, like dentate gyrus. Adult-newborn neurons, in *CX3CR1–/–* mice, show a deficient synaptic integration in the neuronal network and exhibit a diminished amount of dendritic spines. These display some morphological alterations, since mice lacking CX3CR1 protein have a hyperactive, anxiolytic-like, and depressive-like phenotype (101).

Mainly, all the previous remarks are observed in mouse models, but little is known about the consequences of changes in microglia in humans. Interestingly, CX3CL1 concentrations are reduced in the cerebrospinal fluid (CSF) of AD patients compared to control subjects, thus suggesting that variations in CX3CL1 levels might represent a new target to use in inflammation and AD (102). Two recent different publications describe the consequences, in humans, of having homozygous mutations in the colony-stimulating factor 1 receptor (*CSF-1R*) gene expressing a cell receptor essential for the development and maintenance of microglia. The consequences are the presence of abnormalities not only in brain structures, like *corpus callosum*, but also in bones that, in some cases, are overly dense and malformed (103, 104). In the future, it will be interesting to explore possible changes in adult neurogenesis at the dentate gyrus in the autopsy, in the cases of patients with biallelic *CSF-1R* mutations.

## Cellular and Molecular Neuroinflammatory Pathways in Alzheimer's Disease

Neuroinflammatory pathways and microglial cells activation are associated with neuronal ectopic cell cycle activation (105). In particular, microglial activation induced by Aβ oligomers promotes neuronal ectopic cell cycle events (CCEs) via the tumor necrosis factor-alpha (TNF-α) and the c-Jun kinase (JNK) signaling pathways. Hence, administering of non-steroidal anti-inflammatory drugs (NSAIDs) in AD transgenic mice precludes both microglial activation and stimulation of CCE (105, 106). Two analyses report the capability of ibuprofen to alter the advancement of mild AD (107). However, forthcoming AD clinical trials show no effectiveness in mild dementia individuals, probably because these drugs are administered in a late phase of CNS inflammation. Indeed, recent studies designate initial CNS inflammation as an encouraging target to prevent the advancement of the pathology (19).

Today, it is widely accepted that oxidative stress is strongly associated with the inflammation observed in AD (108). In fact, neuroinflammatory processes can act both as cause and as effect of chronic oxidative stress (Figure 2). In this context, microglia play a pivotal role. Proinflammatory microglial activities may be detrimental in AD due to reactive oxygen and nitrogen intermediate species—ROS and RNS, respectively—leading to oxidative stress-induced neuronal death, which could be further exacerbated by chronic stress (109, 110). Cumulative evidence suggests that microglial inflammation-induced oxidative stress in AD is amplified. In contrast, microglial-mediated clearance mechanisms are not functional (43, 110).

TNF-α exerts a key role in this early proinflammatory process observed in preclinical AD as emerges from preclinical studies in animal models of AD (111–114) as well as from human longitudinal studies (21, 113–115). TNF-α is chronically released during the course of AD pathology, likely by activated microglia, neurons, and astrocytes stimulated by increased levels of extracellular Aβ (111). Aβ oligomeric forms activate microglia with anomalous TNF-α-mediated pathways in mouse models (68). Such an atypical stimulation of cerebral innate immunity is responsible for reduced serotonergic tonus, a primary event in depression due to Aβ, a prodromal symptom of AD (70). On the other hand, TNF-α can stimulate γ-secretase activity, which results in an increased synthesis of Aβ peptides and a further increase in TNF-α release (113, 116). It is hypothesized that this auto-amplified loop in the AD brain can contribute to the maintenance of excessive levels of TNF-α, which could then stimulate Aβ synthesis and neuronal loss, also inhibiting microglia phagocytosis of Aβ (Figure 2) (113, 117). Finally, TNF-α significantly contributes to promote insulin resistance and the following cognitive decline in AD (118, 119). Aβ oligomeric forms prompt peripheral glucose intolerance in mice by activating TNF-α signaling in the hypothalamus (120). Multiple studies detected elevated TNF-α levels in both mild cognitive impairment (MCI) and AD (21, 113). Interestingly, Down syndrome cases with preclinical AD show significant links among augmented levels of plasma TNF-α, Aβ accumulation, and the following cognitive deterioration in the subsequent years (115).

TNF-α exerts its activity by binding two distinct high-affinity receptors (TNF-Rs) placed at the cell surface: TNF-RI, ubiquitously expressed apart from erythrocytes, and TNF-RII, whose expression is limited to myeloid cells, endothelial cells, oligodendrocytes, microglia, astrocytes, and subpopulations of neurons (113). The concentrations of the soluble forms of the TNF receptors (sTNF-RI and sTNF-RII) are typically unaltered in CSF and blood of AD patients compared to controls (21). However, both TNF-α and TNF-RI concentrations are increased in postmortem brains of early-stage AD patients (113). MCI subjects present controversial data; longitudinal studies report associations between TNF-R concentrations and the risk of conversion from MCI to AD (21). Notably, the TNF-α receptor complex and its functional proteins are assumed to play a crucial role since they link neuroinflammatory pathways to amyloid deposition process in a chronically damaging and self-perpetuating way (21).

A strong neurobiological link is also found in the AD brain between the deficit of anti-inflammatory cytokines, such as TGF-β1, and the early proinflammatory process observed in preclinical AD (70). TGF-β1 is a neurotrophic factor whose deficit exerts a key role in AD. A selective impairment of TGF-β1 pathway is present in early AD, both in the AD brain (121, 122) and in AD animal models (71, 123, 124). This deficit seems to critically contribute to neuroinflammation in AD brain. TGF-β1 displays both anti-inflammatory and neuroprotective actions (123, 125) and stimulates Aβ clearance by microglia (126). Furthermore, it exhibits a primary role in synaptic plasticity and memory creation processes, thus supporting the path from early to late long-term potentiation (LTP) (127). We should reconsider the relevance of TGF-β1 in neuroinflammation resulting from microglia activation, contributing to reactivate the neuronal cell cycle in the AD brain (128). According to this scenario, the reactivation of the neuronal cell cycle might be assisted by the disruption of Smad-dependent TGF-β1 pathways. Overall, these studies suggest the potential contribution of the deficit of Smad-dependent TGF-β1 pathway to neuroinflammation and cognitive impairment (70).

Moreover, neuroinflammatory phenomena might impair neurotrophin signaling (Figure 2) and interfere with brain-derived neurotrophic factor (BDNF)-induced neuroprotection (129–131).

Finally, neuroinflammation can exert a primary function in AD pathophysiology by interfering with nerve growth factor (NGF) maturation and function. NGF is a neurotrophic factor essential for the survival and homeostasis of basal forebrain cholinergic neurons whose selective degeneration critically contributes to cognitive decline in AD patients (132, 133). Studies in transgenic animal models of AD indicate that the proinflammatory process—initiated before plaque deposition and promoted by soluble Aβ oligomers—leads to an impairment of NGF metabolic pathway characterized by a reduced conversion of the precursor proNGF to the mature NGF (mNGF) as well as by an increased deprivation of mNGF (18, 132, 134). Neuroinflammatory processes promote an overactivation of metalloprotease-9 (MMP-9), as observed in the brains of Down syndrome patients (132), MCI subjects, and AD patients (135). Increased MMP-9 activity would then facilitate the degradation of mNGF, finally compromising mNGF activity in sustaining the trophic dependence of the cholinergic neurons (132). Notably, a strong correlation is present in Down syndrome cases showing preclinical AD among the plasma TNF-α increase, a deficit in NGF maturation (with grown concentrations of proNGF), and an increased degree of cognitive impairment (115). This study substantiates the key contribution of inflammatory markers (i.e., TNF-α) in combination with plasma Aβ_1−42_ levels and increased proNGF levels to better predict the worsening of “latent” AD pathology with the consequential cognitive decline in Down syndrome patients (115). The discovery of an imbalance in the metabolic pathway controlling NGF maturation and degradation in Down syndrome/AD patients provides a platform for the identification of novel biomarker candidates as well for the development of disease-modifying drugs. Therefore, drug discovery processes should be directed in the future to develop new drugs that are able to interfere with early CNS inflammation and, at the same time, rescue neurotrophin signaling (e.g., BDNF, NGF, TGF-β1) in the AD brain.

## Targeting Neuroinflammation in Alzheimer's Disease: Evidence From Animal Models

Among the different mediators of inflammation explored, TNF-α mediates proinflammatory processes in various ND including AD (136). In normal conditions, TNF-α from glial cells modulates homeostatic activity-dependent regulation of synaptic connectivity (137). On the other hand, this cytokine mediates the disrupting effects of Aβ on LTP in experimental AD. Accordingly, mutant mice lacking TNF receptor type 1 exhibit normal LTP following Aβ application and similar results are obtained with the use of anti-TNF agents including the monoclonal antibody infliximab and thalidomide, which also inhibits TNF-α production (138). Generally, several studies indicate that blocking the TNF-α pathway in AD models is associated with: (I) improvement in memory decline, as tested in different behavioral tests evaluating cognitive function; (II) reduction in immunohistochemical and histopathological markers like formation of Aβ plaques and NFT; and (III) reduction in the number of microglial cells in the AD brain (139).

Similarly to TNF-α, also the proinflammatory cytokine IL-1β mediates the synaptotoxic effects of Aβ peptide (140). Indeed, the interleukin-1 receptor antagonist (IL-1Ra) is able to reverse synaptic plasticity alteration triggered by the administration of the 40-amino acid-long Aβ peptide (Aβ_1−40_) (141). However, the role of ILs in AD pathogenesis is far more complex since some exert proinflammatory while others exert anti-inflammatory actions. In this frame, it is worth mentioning IL-12 and IL-23 which are increased in CSF in both AD and MCI (142, 143). Notably, genetic ablation of IL-12 and IL-23 or therapeutic approaches directed against IL-12 and IL-23 signal reduce the AD-like pathology, including histopathological and behavioral changes, making them attractive targets for the treatment of AD (144). On the other hand, IL-10 seems to play a protective role since delivery of this cytokine via adeno-associated virus leads to markedly decreased microgliosis and astrogliosis as well as reversed cognitive impairment in transgenic AD mice (145), although the use of a different adeno-associated virus approach generates a different outcome (146).

There is a growing interest on the role of complement and microglia in AD pathology (147). Microglia cells have prominent functions in complement-mediated synaptic pruning, in the postnatal period (148, 149). It is hypothesized that an inappropriate reactivation of this mechanism later in life could result in synapse loss, thus facilitating the progression of ND (150). In this frame, C1q, which mediates the toxic effects of Aβ oligomers on LTP, is increased in synaptic connections before plaque deposition, and inhibition of C1q, C3, or the microglial complement receptor CR3 diminishes phagocytic microglia, resulting in protection against synapse loss (151).

Investigations conducted in transgenic AD mice also address the effects of NSAIDs on amyloid load and inflammation (152). These studies suggest that NSAIDS not only exert neuroprotection through the suppression of inflammatory events but also reduce early amyloid pathology by mechanisms that remain unclear (153). Of note, two selective cyclooxygenase-2 (COX-2) inhibitors are found to be effective in rescuing LTP impairment by synthetic soluble Aβ_1−42_, whereas the same effect is not achieved with the cyclooxygenase-1 (COX-1) inhibitor piroxicam (154). Similarly, ibuprofen prevents early memory decline in AD model, and this effect is associated with activation of hippocampal plasticity-related genes (155). Overall, these studies indicate that NSAIDs exert neuroprotection and prevent memory decline through the modulation of multiple neuronal pathways (156).

## Biomarkers of Neuroinflammation in Alzheimer's Disease

Most of the failed AD clinical trials—including trials investigating anti-inflammatory compounds—did not assess any biological *in vivo* identification of AD-related pathomechanistic alterations, thus preventing proof of mechanisms (157) and including a percentage of subject displaying non-AD pathophysiology (158). Therefore, robust biomarkers–drug codevelopment pipelines are strongly recommended for next-generation clinical trials (159).

### Fluid Biomarkers of Neuroinflammation in Alzheimer's Disease

Modifications of the concentrations of several cytokines (160–164) and other inflammatory biomarkers associated with either microglia—e.g., soluble TREM2 (sTREM2), monocyte chemoattractant protein-1 (MCP-1), and YKL-40 (165–168)—or astroglia, e.g., YKL-40 (161), are extensively investigated in AD patients. These alterations, potentially, reflect the inflammatory mechanisms within the CNS coupled with the neurodegenerative pathways (11, 166). A recent meta-analysis reports higher concentration of YKL-40, sTREM2, MCP-1, and TGF-β in the CSF of AD patients compared to controls (160). In particular, robust evidence from several studies focus on CSF YKL-40 that shows a fair classificatory capability in differentiating between AD individuals and controls as well as in predicting the progression from the asymptomatic to later prodromal and dementia stages (166–168). However, its function in differentiating subjects with AD and other dementia remains controversial since neuroinflammation seems to be associated with neurodegeneration *tout court* and not with specific neurodegenerative pathways (12, 169).

The clinical meaning of inflammatory biomarkers in blood needs to be elucidated, as they might represent low-invasive and low-cost screening tools of cerebral inflammatory activity during the early asymptomatic stages of AD (170–172). The main issue concerning the peripheral measurements of inflammatory biomarkers is that they may not directly reflect brain neuroinflammation (163). Nonetheless, IL-6 and IL-1β concentrations are significantly higher in AD compared to cognitively normal controls in four meta-analysis (160–163). IL-1β is a key molecule participating in the inflammatory response, cell proliferation, differentiation, and apoptosis. Some evidence suggest that IL-1β is produced and secreted by microglia cells in response to Aβ deposition, thus resulting in chronic neuroinflammation and, eventually, neuronal disruption, dysfunction, and neurodegeneration (173, 174). A negative correlation between CSF concentrations of this cytokine and cognitive scores has also been described in AD (175). IL-6 levels are associated with the severity of cognitive decline as assessed by Mini-Mental State Examination (MMSE) scores (161). Notably, peripheral IL-6 concentrations positively correlate with the cerebral ventricular volumes (176) and with matched CSF samples (177) in AD. The peripheral modifications of IL-6 levels could begin in the prodromal phase of AD; indeed, a recent meta-analysis highlights the greater IL-6 concentrations in MCI subjects compared to controls (160). In line with these findings, a longitudinal study reports the association of elevated plasma IL-6 levels with a greater risk of cognitive decay, at 2-year clinical follow-up. Other cytokines emerging as candidate peripheral inflammatory biomarkers are IL-2, IL-12, IL-18, and TGF-β (160–163).

Overall, these studies have several biases to consider. First, the risk of misdiagnosis is high since AD and MCI diagnoses are mainly clinical based in the majority of the studies lacking the necessary biomarker information [e.g., cerebral amyloid-positron emission tomography (PET) uptake or CSF Aβ_1−42_ measurements]. This means that at least 20–25% of the AD patients and MCI subjects enrolled in the previous studies do not have cerebral amyloid deposition (6). Moreover, these studies are cross-sectional without an appropriate follow-up, and this could lead to incorrect MCI diagnosis. Indeed, the clinical picture of MCI is heterogeneous not only with a 10–15% annual rate of developing AD (178) but also with a consistent proportion of individuals who recover, remain stable, or develop ND other than AD (179). In addition, the MCI classification (e.g., amnestic or non-amnestic), which significantly impacts clinical outcome (8, 179, 180), is inadequately specified in most of the studies. Furthermore, data regarding comorbidities—such as cerebrovascular diseases, coronary diseases, atrial fibrillation, periodontitis, and diabetes or concomitant drugs (e.g., non-steroidal inflammatory medications, corticosteroids, statins) that can significantly modify peripheral inflammatory biomarkers—have been rarely reported. For instance, persistent higher plasma levels of IL-1β and IL-6 are observed in relation to cardiovascular diseases as well as atherosclerosis (181, 182). Other potential biases include technical issues: detection methods (e.g., ELISA kits) for inflammatory biomarkers in biological fluids are consistently different among studies as well as sample handling approaches [e.g., measurements on different fluids matrix (plasma or serum) and storage protocols].

In conclusion, neuroinflammation is certainly a relevant pathophysiological mechanism of neurodegeneration in AD. However, we still lack reliable inflammatory biomarkers to be used in a screening context of use. In essence, sTREM2, MCP-1, IL-6, TGF-β, and, particularly, YKL-40 are interesting novel inflammatory CSF biomarkers, but they cannot be proposed in detecting the early asymptomatic phases of AD, as it would be altered with disease-modifying treatments. Prospective observational studies enrolling large cohorts of participants with accurate clinical and biomarker-based characterizations are needed to identify potentially effective inflammatory blood-based biomarkers of AD.

### PET Radiotracers Targeting Neuroinflammation in Alzheimer's Disease: State-of-the-Art on Human Studies

There are several genetic association studies highlighting a key role of neuroinflammation in AD by demonstrating the occurrence of specific genetic variations related to immune response in patients with ND including AD (183). As a direct consequence, the possibility of tracking the regional evolution of neuroinflammation and imaging non-invasively the neuroinflammatory process in AD patients opens up exciting novel opportunities to monitor disease progression and, eventually, to explore immune-therapeutic strategies to prevent or decelerate disease progression.

It is interesting to note that it could be possible to assess the neuroinflammatory status by conventional [^18^F]-fluorodeoxyglucose (FDG)-PET, provided that the whole uptake curve is studied (184). Neuroinflammation can be measured more specifically using targeted radio-ligands for PET imaging—that allow accomplishing the regional *in vivo* exploration of neuroinflammation—like [^11^C]-PK11195 (185). A number of studies show alterations in [^11^C]-PK11195 binding in AD and several other ND (186–189), Parkinson's disease (190), and progressive supranuclear palsy (189, 191), and the distributions of [^11^C]-PK11195 found in these studies are akin to the well-known distribution of neurodegeneration (e.g., posterior cortical regions in AD). However, one should also note that translocator protein (*TSPO*) gene polymorphisms can greatly affect binding affinity (192), and TSPO expression is not circumscribed to activated microglia, which can also occur on astrocytes, or endothelial cells (193). In this context, a number of novel TSPO-specific PET radiotracers are currently available, both carbon-11 (i.e., [^11^C]-PK11195, [^11^C]-PBR28) and fluoroine-18 labeled [e.g., [^18^F]-GE-180, [^18^F]-DPA-714, and [^18^F]-PBR06], typically used in preclinical investigations (194, 195). In addition, a very recent tracer named [^18^F]-FEPPA is able to provide a high potential for TSPO-PET in humans (192).

Interestingly, microglia activation is only one (albeit important) part of the chain of events that eventually lead to neuroinflammation and that can potentially be imaged with even more specific tracers. For example, protein misfolding, aggregation, and accumulation may trigger glial response and, therefore, neurotoxicity. To date, the causal relationships between neuroinflammation and other pathogenetic mechanisms of AD is not elucidated yet. PET radiotracers can represent a suitable tool for untangling these dynamics along the roadmap of discovering new targets for anti-inflammatory disease-modifying strategies. In this context, a number of specific PET tracers can target protein aggregates in the brain. For example, the [^11^C]-Pittsburgh compound-B ([^11^C]-PIB) is able to bind Aβ fibers (186, 190). Aβ can also be imaged through, e.g., (^18^F)-labeled derivatives like [^18^F]-Florbetaben, [^18^F]-Florbetapir, and [^18^F]-Flutemetamol (196). In addition, hyperphosphorylation and abnormal aggregation of tau, which is crucial to neuronal activity, can be imaged using definite tracers, using specific ligands: T807, Flortaucipir as well as the phenyl/pyridinyl-butadienyl-benzothiazole/benzothiazolium derivative PBB3 (197, 198). Additionally, tracers [^18^F]-FA and [^18^F]-EFA (analogs of 2-fluoroacetate, which can be utilized to inhibit glial cell metabolism) are able to selectively enter the metabolic compartment (199) and may, therefore, be promising candidates for evaluating glial metabolism when thinking of astrocytic response.

Finally, there are other molecular targets that can offer a more exhaustive depiction of *in vivo* neuroinflammation (200). For example, the cyclooxygenase (COX) enzyme is involved in both inflammation and generation of proinflammatory mediators. In this context, COX-1 radioligands, like [^11^C]-KTP-Me, show promising results in AD animal models (201). In addition, the cannabinoid receptor type 2 (CB2R) is subject to upregulation in activated microglia in various ND (202), possibly in conjunction with a neuroprotective effect (203), and postmortem studies emphasize the potential of compounds like [^11^C]-RS-016, which show high specific binding (204). This is emphasizing the role of CB2R as an additional potential target for PET imaging of neuroinflammation, in humans. Further encouraging targets examined in preclinical examinations are the purinergic receptor P2X7 ([^11^C]-GSK1482160) (205) and the adenosine receptor A2AR (i.e., [^11^C]-TMSX).

## Why Did Anti-inflammatory Therapy Fail in Alzheimer's Disease?

### Clinical Trials of Anti-inflammatory Drugs in Alzheimer's Disease

NSAIDs have long been hypothesized to play a protective role in AD. This assumption is reinforced by several cohort analyses. A recent meta-analysis including 16 investigations demonstrate that present or previous utilization of NSAIDs is linked to a decreased relative risk of AD (0.81; 95% confidence interval, 0.70–0.94) (206). Despite the observational epidemiological data suggesting a protective effect of NSAIDs and the evidence for a biologically plausible role for anti-inflammatory treatment, all placebo-controlled trials of a wide range of anti-inflammatory agents (NSAIDs, corticosteroids, and others) in both mild-to-moderate AD patients (Table 1) and MCI subjects (Table 2) are negative. Studies in cognitively normal subjects at risk of developing AD are also negative (Table 3). The first, large primary prevention study of naproxen and celecoxib [Alzheimer's Disease Anti-inflammatory Prevention Trial (ADAPT)] has been prematurely interrupted for cardiovascular safety concerns after the enrollment of 2, 528 subjects in the study and their treatment for a median time of 2 years. The study is not able to support the hypothesis that either drugs could postpone AD beginning in adults with a family history of dementia (227). A subsequent 2-year, primary prevention trial [Impact of Naproxen Treatment in Pre-symptomatic Alzheimer's Disease (INTREPAD)] has been used to compare the effects of naproxen and placebo on the Alzheimer Progression Score (APS) in 195 cognitively normal older persons with a positive family history of AD (226). Over time, the APS scores progressively increase to a similar extent in both study groups, thus suggesting that naproxen does not provide any benefit over placebo in slowing the progression of presymptomatic AD.

**Table 1:** Double-blind, randomized, placebo-controlled trials using anti-inflammatory drugs in mild-to-moderate AD patients.

**Drug**	**Dose (mg/day)**	**Therapy duration (month)**	**Number of patients**	**Main effect**	**References**
Celecoxib	400	12	285	Neutral	(207)
Celecoxib	400	12	425	Neutral/detrimental	(208)
Dapsone	100	12	201	Neutral	(209)
Diclofenac	50	6	41	Beneficial	(210)
Hydroxychloroquine	200–400	18	168	Neutral	(211)
Ibuprofen	800	12	132	Neutral	(212)
Indomethacin	100–150	6	44	Beneficial	(213)
Indomethacin	100	12	51	Beneficial	(214)
Naproxen	440	12	351	Neutral	(215)
Nimesulide	200	3	40	Neutral	(216)
Prednisone	10	12	138	Neutral/detrimental	(217)
Rofecoxib	25	12	351	Neutral/detrimental	(215)
Rofecoxib	25	12	692	Neutral	(218)
Tarenflurbil	800–1,600	12	210	Neutral	(219)
Tarenflurbil	1,600	18	1,684^*^	Neutral/detrimental	(220)
Tarenflurbil	1,600	18	840^*^	Neutral	(221)

* Patients with mild AD.

*AD, Alzheimer's disease*.

**Table 2:** Double-blind, randomized, placebo-controlled trials using NSAIDs in MCI individuals.

**Drug**	**Dose (mg/day)**	**Therapy duration (month)**	**Number of patients**	**Main effect**	**References**
Celecoxib	200–400	18	88*	Beneficial	(222)
Rofecoxib	25	48	1,457	Detrimental	(223)
Triflusal	900	13	257	Neutral/beneficial	(224)

* Subjects with age-associated memory decline.

*MCI, mild cognitive impairment; NSAIDs, non-steroidal anti-inflammatory drugs*.

**Table 3:** Double-blind, randomized, placebo-controlled primary prevention trials using NSAIDs in AD.

**Drug**	**Dose (mg/day)**	**Therapy duration (month)**	**Number of patients**	**Main effect**	**References**
Celecoxib	400	24	2,528	Neutral/detrimental	(225)
Naproxen	220	24	160	Neutral	(226)
Naproxen	440	24	2,528	Neutral/detrimental	(225)

*AD, Alzheimer's disease; ADAPT, Alzheimer's Disease Anti-inflammatory Prevention Trial; NSAIDs, non-steroidal anti-inflammatory drugs*.

### Stage-Dependent Neuroinflammatory Process in the Alzheimer's Brain

**Figure 3:**
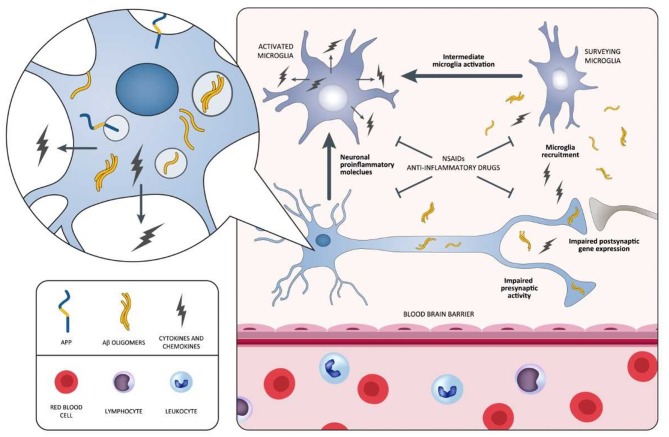
Schematic representation of neuroinflammatory process occurring during the early stages of the AD pathology and potential points of attack of NSAIDs and anti-inflammatory drugs. In this process, neurons surrounded by Aβ oligomers release proinflammatory cytokines triggering the intermediate activation of microglia and their mobilization toward Aβ-burdened neurons. Both Aβ-burdened neurons and activated microglia are responsible for a disease-aggravating process in which the release of proinflammatory cytokines and chemokines predominates. NSAIDs and anti-inflammatory drugs may be potentially effective during this early inflammatory phase, antagonizing the aggravating activity of proinflammatory mediators. Selective agents stabilizing microglia may also be effective in attenuating the inflammatory process. Aβ, amyloid beta; AD, Alzheimer's disease; NSAIDs, non-steroidal anti-inflammatory drugs.

In spite of emerging epidemiological evidence, all large, longstanding, randomized, placebo-controlled investigations aiming at attenuating cerebral inflammation in AD display negative outcomes. The fact that anti-inflammatory therapies are not able to safeguard patients with overt dementia has been debated. Actually, a trial recruiting MCI individuals highlights that rofecoxib could rush the conversion to AD (223). Moreover, a primary prevention study involving celecoxib and naproxen in cognitively healthy elderly individuals showing family history of AD has been terminated in advance due to the existence of negative or harmful effects generated by the drugs (225, 228). Additional longstanding, controlled studies examining anti-inflammatory drugs, including tarenflurbil in mild AD patients (220), prednisone (217), and celecoxib (208) in mild-to-moderate AD patients, report the presence of detrimental consequences vs. placebo. NSAIDs negative and/or harmful effects, documented in AD, MCI, as well as in the stages preceding AD, are apparently in conflict with epidemiological data indicating diminished AD incidence after sustained treatment with NSAIDs. This is potentially related to the different impact of the disease stages on NSAIDs exposure. In this context, two different inflammatory responses in the AD pathophysiological process are assumed to exist: (I) one, at the early preclinical stage, with a predominantly proinflammatory component that is amenable to therapy; (II) another, at a later clinical stage, with predominantly innate/adaptive immune reactions not responsive to anti-inflammatory therapy (19). During the early inflammation stage, neurons stimulated by Aβ initiate the inflammatory process, and then, they induce intermediate microglia cells activation and their recruitment around Aβ-burdened neurons. Both neurons and microglia elicit a process exacerbating the disease characterized by the release of proinflammatory mediators (cytokines and chemokines) (Figure 3). The inflammatory immune response of the late plaque-associated stage involves different processes, including full microglial activation, microgliosis, and CNS invasion by peripheral monocytes. Both microglia and monocytes participate in phagocytic activities to eradicate toxic Aβ oligomers and, probably, cellular debris (19). This assumption is in line with data from the Rotterdam (229), the Cache County (230), and the US Veterans (231) observational studies. The above-mentioned analyses emphasize the lack of protection following 2-year NSAID exposure before dementia onset. In case the timing of exposure defines whether NSAID administration is beneficial or harmful, then the negative results of the previously mentioned studies (ADAPT and INTREPAD) are not unexpected, given that the timing of exposure of the participants to NSAIDs was restricted (2 years). On this basis, NSAIDs might be useful for AD prevention when their administration occurs years before the usual onset age; however, when used later in life, they might increase the risk of disease. We cannot exclude the possibility that (I) the majority of the advantageous effects of NSAIDs, documented from epidemiological studies, may originate from different types of bias (232), and (II) actually, there is no established impact of NSAIDs on AD prevention or treatment (233).

Alector/Abbvie claim the generation of a monoclonal antibody (AL002) that binds and activates TREM2. AL002 entered its first phase 1 trial in 51 healthy adults and 16 AD patients (234). Alector is also starting its first trial of the anti-CD33 antibody, AL003. The microglial receptor CD33 opposes the effects of TREM2 signaling and may present a more amenable target because it would be inhibited rather than activated. In the first phase of the trial, 42 healthy adults will receive a single treatment of either placebo or one of seven different AL003 doses. The second, multiple-dose phase will enroll 12 AD patients, two of whom will receive placebo (234).

Of note, other drugs targeting neuroinflammation to treat AD are being developed and underwent clinical testing. XPro1595 is currently undergoing phase 1b clinical trials. Other examples are GC021109 and NP001. XPro1595 is a variant of TNF-α that forms heterotrimers with native soluble TNF-α and prevents its interaction with the type 1 TNF-α receptors (235). Unlike other non-selective TNF-α inhibitors, XPro1595 does not suppress innate immunity or myelination mediated by type 2 receptors (236). Differently from etanercept, long-term treatment with XPro1595 does not suppress hippocampal neurogenesis, learning, and memory in adult mice (237). In 5xFAD mice, twice-weekly subcutaneous administration of XPro1595 for 2 months reduced brain amyloid deposition and immune cell infiltration, and improved synaptic function (238). In young TgCRND8 mice, continuous subcutaneous infusion of XPro1595 for 1 month prevented brain amyloid deposition and normalized hippocampal neuron synaptic function (239). In 3xTg mice, intracranial administration of XPro1595 reduced amyloid pathology (240). In aged wild-type rats, intracranial infusions of XPro1595 for 6 weeks reduced microglia activation and improved synaptic function and cognition (241). A 12-week, open-label, phase 1b study of XPro1595 (weekly injections of 0.03, 1.0, or 3.0 mg/kg) is ongoing in 18 mild-to-moderate AD patients (NCT03943264). Participants were requested to have a positive amyloid test and evidence of peripheral inflammation [elevated blood C-reactive protein (CRP)]. Biomarkers of neuroinflammation in blood and CSF (CRP, TNF-α), IL-1β, and IL-6 are being measured.

GC 021109 targets microglial cells by binding the P2Y6 receptor, a metabotropic G-protein-coupled receptor, whose natural ligand is adenosine diphosphate, a metabolite of ATP. Astrocytes release ATP in response to the presence of Aβ aggregates and P2Y6 signaling is thought to be involved in shifting the phenotype of microglia, which tend to surround amyloid plaques, from patrolling to phagocytic (242). GC 021109 has been reported in the biotech press to stimulate both microglial phagocytosis and inhibit microglial release of proinflammatory cytokines such as IL-12; however, this information is not published in the peer-reviewed literature. A phase 1a study in 44 healthy volunteers has been carried out in 2015 (NCT02254369), and a 4-week, phase 1b study in 39 mild-to-moderate AD was completed in 2016 (NCT02386306). However, no results were reported.

NP001 is a pH-adjusted intravenous formulation of purified sodium chlorite. Within monocytes/macrophages, chlorite is converted into taurine chloramine that downregulates the nuclear factor kappa-light-chain-enhancer of activated B cells (NF-κB) expression and inhibits production of proinflammatory cytokine IL-1β. These mechanisms of downregulation transform inflammatory monocytes/macrophages from a proinflammatory to a basal phagocytic state. NP001 has been tested in patients with amyotrophic lateral sclerosis (243). A small study planned to be carried out in 14 mild-to-moderate AD patients (NCT03179501) was interrupted in 2018 for poor enrollment.

### Preliminary Evidence of a Potential Biological Effect of NSAIDs

Profiling molecular pathways related to ND is expected to reveal novel pathways for therapeutic agents. In this context, inflammation represents a primarily involved pathway (12, 19). Interestingly, a meta-analysis including 175 studies reports changes in several inflammatory biomarkers (IL-6, CRP, and TNF-α) in AD (161). Another meta-analysis including nine longitudinal studies shows a protective effect by NSAIDs against AD progress (244). Changes in the concentrations of blood (serum) inflammatory proteins—including IL-6, CRP, and TNF-α–define a serum-based proteomic signature potentially useful for AD diagnosis (245–247). Hence, according to the literature, anti-inflammatory compounds might be employed as therapeutic agents in AD and others ND. In this regard, a novel model based on PM for targeted NSAIDs therapy to specific AD patients is recently proposed by O'Bryant and colleagues. In particular, they determine whether a blood proteomic companion diagnostic (CDx) is able to predict response to NSAID treatment (248). The analysis of the proteome in plasma samples from the Alzheimer's Disease Cooperative Studies (ADCS) anti-inflammatory clinical trial, including 1-year administration of rofecoxib (25 mg once daily), naproxen (220 mg twice daily), or placebo (*N* = 351) (215)—indicates that an overall NSAID-general CDx is accurate in detecting treatment response with 87% accuracy. Drug-specific companion diagnostics—Rofecoxib-CDx and Naproxen-CDx—generate a very high degree of accuracy in both rofecoxib (98%) and naproxen (97%) arms (234). This is a relevant example of direct evidence for a precision medicine-based model to address AD treatment via the creation of CDx-driven therapeutics.

### Preliminary Evidence of a Potential Biological Effect of Monoclonal Antibodies Selectively Targeting Aβ Protofibrils

In the last 20 years, a rising body of experimental studies has indicated that soluble Aβ protofibrils are more synaptotoxic than insoluble Aβ plaque cores. For instance, the former display higher rates of synapse structure impairment, including LTP, than plaques (249–251). Of note, solubilization of Aβ plaque cores is strictly related to the release of smaller Aβ species, such as dimers, and downstream increase in synaptotoxicity (252). Therefore, it is argued that prefibrillar Aβ_1−42_ assemblies, rather than monomers or dimers, are the proximate mediators of Aβ toxicity (253).

With regard to CNS immune resident cells, growing evidence indicates that small soluble Aβ_1−42_ protofibrils are the main trigger of microglial activation. Indeed, experimental models of AD indicate that both microglia and astrocytes display not only a high sensitivity to Aβ structure (16, 17, 33, 68, 86) in the internalization process but also emphasize their greater affinity for soluble Aβ protofibrils than mature insoluble fibrils (254, 255). In this context, it is reported that small soluble Aβ_1−42_ protofibrils, rather than fibrils, can induce microglial activation, as reflected by increased cerebral levels of TNF-α (255).

The murine monoclonal antibody mAb158 displays a 1, 000-fold higher selectivity for protofibrils [N-terminal (1–16) of the Aβ sequence] vs. monomers and 10–15 times more efficient binding to protofibrils vs. fibrils (256, 257).

Several studies, employing mAb158, suggest that astrocytic Aβ uptake depends on size and/or composition of Aβ aggregates, since astrocytes, if possible, engulf oligomeric Aβ over its fibrillar aggregation states (258).

Recent trials conducted in mice models of AD demonstrate that the antibody significantly slows down Aβ accumulation in astrocytes reducing the downstream Aβ-induced neuronal toxicity (256). The authors argue that their results provide a strong evidence for astrocytes to play a key mechanistic role in anti-Aβ immunotherapy.

The phase 2 preliminary results regarding the humanized IgG1 monoclonal version of mAb158 [BAN2401 (Biogen, Eisai Co., Ltd./BioArctic Neuroscience AB)]^1^ (259) show that BAN2401 significantly reduces Aβ-PET standardized uptake value ratio (SUVr) as well as CSF neurogranin, p-tau, and neurofilament light chain protein levels over a 18-month clinical trial (260, 261).

## Exercise as an Anti-inflammatory Therapy in Alzheimer's Disease

Acute, unaccustomed exercise (i.e., of an unusual duration and/or intensity) can increase oxidative stress and act as a proinflammatory stimulus (262, 263). However, this response is attenuated when exercise is performed regularly, with strong evidence actually supporting that “chronic” exercise upregulates an endogenous systemic anti-inflammatory response (16).

Large cohort studies indicate that higher levels of physical activity are inversely associated with inflammatory biomarkers, for instance CRP (264, 265). There is meta-analytical evidence that regular physical exercise can reduce inflammation-related biomarkers (e.g., CRP, TNF-α) in middle-aged and older adults (266, 267), these benefits being also present in individuals with cognitive impairment (268). Animal research indicates that the anti-inflammatory effects of exercise can also reach the brain tissue. Physical exercise training results in an enhanced anti-inflammatory status—as reflected by an increased expression of anti-inflammatory cytokines including IL-10β coupled with the decrease in proinflammatory cytokines (including TNF-α)—at the hippocampus level in a rat model of AD (269). Chronic exercise also promotes a conversion of the microglia from the proinflammatory (M1) to the anti-inflammatory (M2) phenotype in different rodent models of disease, including AD (269–272).

Although the mechanisms underlying exercise anti-inflammatory effects remain to be clearly elucidated, several pathways are currently proposed. Notably, contracting muscles act as an endocrine organ by releasing myokines (i.e., cytokines and other small peptides) to the bloodstream, which, in turn, induce numerous health benefits (such as a decrease in inflammation) at the multisystemic level, including the brain (273, 274). Muscle-derived IL-6 promotes the systemic production of anti-inflammatory cytokines (IL-1Ra, IL-10) and downregulates the expression of proinflammatory cytokines (TNF-α, IL-1β) (275). Other proposed mechanisms include exercise-induced reductions in adiposity (which, especially visceral fat, contribute to systemic inflammation), on the one hand, and increases in vagal tone, on the other hand, through the cholinergic anti-inflammatory pathway, an evolutionarily ancient circuit that modulates immune responses and the progression of inflammatory diseases (276, 277). In conclusion, given the documented relevance of inflammation in most ND (169), there is strong biological rationale to support that exercise might serve as a coadjuvant therapeutic strategy against such conditions.

## Perspectives: Precision Medicine for Targeting Neuroinflammation

### General Overview on Precision Medicine

**Figure 4:**
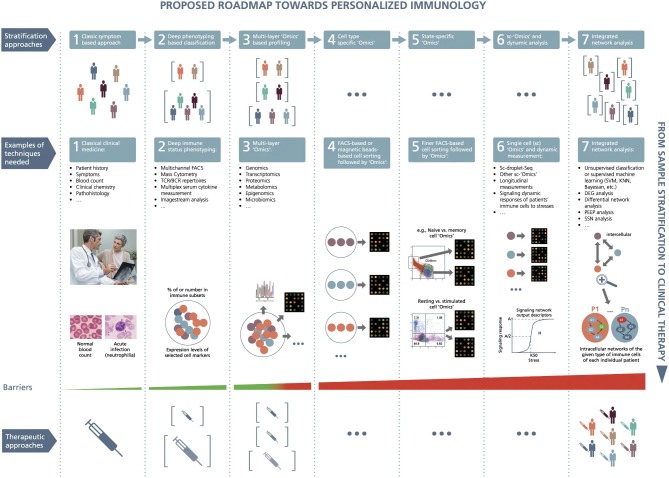
A roadmap proposed toward personalized immunology. There exist both horizontal and vertical roadmaps toward personalized immunology. Vertically, to translate sample stratification to clinical therapies, it is necessary to utilize the state-of-the-art “Omics” analysis and network integration approaches to stratify patients into subgroups and then implement personalized therapeutic approaches to treat individual patients, which needs to overcome various types of barriers at different steps. Horizontally, it might be necessary to go through at least seven steps to enable personalized immunotherapies: (1) classic symptom-based approach, (2) deep phenotyping approach, (3) multilayer “Omics”-based profiling, (4) cell-type-specific “Omics,” (5) state-specific “Omics,” (6) single-cell “Omics” and dynamic response analysis of immune cells, and (7) integrated network analysis. Under the first layer (the so-called stratification layer), different colors of patients indicate individual patients with different cellular and/or molecular profiles, while brackets represent patient subgroups; under the second layer (the so-called technique layers), different small circles with distinct colors indicate different immune cells, while big circles represent patient (sub)groups; under the technique layers, the snapshot of microarray representing either microarray-based or RNA-seq-based transcriptome analysis; under the third layer (the so-called therapeutic layer), the syringes with different colors or tonalities indicate different therapeutic approaches; P1,…, P*n* at step 7 designate different patients; G1, G2, G3, and G4 represent different genes, the arrows between them representing regulatory relationships. DEG, differential expression gene; FACS, fluorescence-activated cell sorting; KNN, K-nearest neighbors; PEEP, personalized expression perturbation profile; sc, single-cell; SSN, sample-specific network; SVM, support vector machine; TCR/BCR, T-cell receptor/B-cell receptor. From Delhalle et al. (284). Copyright© 2018, Springer Nature. Reprinted with permission from Creative Commons CC BY. S.

The official launch of the US Precision Medicine Initiative (PMI), in 2015 (https://obamawhitehouse.archives.gov/precision-medicine), by the US President Obama followed by the National Institutes of Health (NIH) development of the US PMI Cohort Program (PMI-CP) (278) and the creation of the US “*All of Us Research Program*” (available at https://allofus.nih.gov/) are contributing to make precision medicine one of the key topics in biomedical research, worldwide. These facts support the evolution of Medicine from the outdated “one-size-fits-all” paradigm—according to which treatments are conceived for the “average patient”—to the search for comprehensive and accurate stratification of individuals and future individually tailored therapeutic modalities and targeted therapies (279). Indeed, genetic and biological heterogeneity among individuals sharing the same clinical features (so-called clinical syndrome) is highly frequent in polygenic, multifactorial diseases with complex and non-linear pathophysiological dynamics, such as cancer and AD. In this regard, it is acknowledged that the adaptive and innate immune systems are characterized by enormous individual heterogeneity that accounts for the subject-specific response to vaccines and other immunomodulatory therapies (280–282). As a result, some drugs, regularly administered, can be of benefit only to a restricted subset of patients; other drugs might even have detrimental effects to some definite ethnic groups (283). Hence, the identification of the molecular/cellular and environmental factors indicating the presence and the type of reaction of a single AD patient to a specific therapy is crucial (284). The shift to individualized therapies and targeted treatments needs exploratory, unbiased, high-throughput, integrative, large-scale analyses of the features of the cohort's individuals with the disease (279, 284, 285). Cohorts stratified according to different multimodal-throughput technological platforms (“omic” sciences)—*via* systems biology (285, 286)—and different neuroimaging modalities—via systems neurophysiology (285)—can be assimilated in the disease modeling to stratify and predict AD patient subgroups (279, 285). Both systems biology and neurophysiology enable to perform a holistic, systemic exploration of complex interactions in biological systems, thus allowing an overview of cells/groups of cells, tissues, organs, organisms, and populations at multiple scales. High-throughput, integrative approaches permit to recover exhaustive biological information, supported by advanced powerful bioinformatics. This will enable the inclusive integration of both multiomic and clinical data to attain fast and significant interpretation. Precision medicine capitalizes on these theoretical and technological advancements (287). Particularly, the integration of the “omics” and the development of the “multiomic” disciplines—such as proteogenomics, whereby the involved technologies are next-generation sequencing and mass spectrometry—seem to be able to offer a substantial support for accurate phenotype prediction, individualized patient management, and precision medicine (288, 289). Establishing precision medicine needs the implementation of a network of integrated disciplines and methods including the “omic” sciences, neuroimaging modalities, cognitive examinations, and clinical features. All these congregate toward many domains investigated using the systems theory approach (290). This allows the development of models explaining all systems levels—explored via systems biology and systems neurophysiology—and the different categories and scales of spatiotemporal data describing the complexity and clinical heterogeneity of any polygenic disease belonging to any medical fields, from oncology, to immunology (284) (Figure 4), to neurology (285, 291, 292). Precision medicine aims at ameliorating the efficacy of prevention strategies and therapies using customized treatments tailored on the individual's “biological make-up” (285, 291, 292), based on the “P4 Medicine” (*P*4*M*) framework (293). To safeguard the rapid and full expansion of precision medicine in AD, the international Alzheimer Precision Medicine Initiative (APMI) and its own Cohort Program (APMI-CP) (available at https://www.apmiscience.com/)—thematicall associated with the US PMI and the US “*All of Us Research Program*”—are currently established and operational (279). In this connection, a therapeutic plan based on immune/inflammation modulation for a subset of AD and associated dementias is currently ongoing within the “Korean AD Research Platform Initiative Based on Immune-Inflammatory Biomarkers” (K-ARPI) (294).

## Conclusions

Systems theory/biology-based studies are needed to untangle the spatiotemporal dynamics of neuroinflammation and its related subcomponents. Biomarkers simultaneously tracking different molecular pathways (body fluid matrixes) along with brain neuroinflammation endophenotypes (neuroimaging markers) can untangle key temporal–spatial dynamics among glia, neuroinflammation, and other AD pathophysiological mechanisms. Implementing this approach will be necessary to fill the gap in the understanding of whether neuroinflammation represents a direct pathophysiological or compensatory mechanism or both, along the AD continuum. According to this assumption, a new pathway (mechanism)-based pharmacological model—intended to establish effective and functional biomarker-guided targeted and tailored treatments for preventive and neuroinflammation-freezing strategies—needs to be developed.

## Author Contributions

HH, AV, and SL designed the concept of the manuscript, provided supervision and assisted with the writing and content of the manuscript. AV and SL assisted in the preparation of the figures. All authors provided their contribution in writing the manuscript, critically reviewing the completed manuscript, and approved the submitted version of the manuscript.

### Conflict of Interest

HH is an employee of Eisai Inc. and serves as Senior Associate Editor for the Journal Alzheimer's & Dementia; during the past 3 years he had received lecture fees from Servier, Biogen, and Roche; research grants from Pfizer, Avid, and MSD Avenir (paid to the institution); travel funding from Eisai, Functional Neuromodulation, Axovant, Eli Lilly and company, Takeda and Zinfandel, GE-Healthcare, and Oryzon Genomics; consultancy fees from Qynapse, Jung Diagnostics, Cytox Ltd., Axovant, Anavex, Takeda and Zinfandel, GE Healthcare, Oryzon Genomics, and Functional Neuromodulation; and participated in scientific advisory boards of Functional Neuromodulation, Axovant, Eisai, Eli Lilly and company, Cytox Ltd., GE Healthcare, Takeda and Zinfandel, Oryzon Genomics, and Roche Diagnostics. He is coinventor in the following patents as a scientific expert and has received no royalties: *In Vitro* Multiparameter Determination Method for the Diagnosis and Early Diagnosis of Neurodegenerative Disorders Patent Number: 8916388; *In Vitro* Procedure for Diagnosis and Early Diagnosis of Neurodegenerative Diseases Patent Number: 8298784; Neurodegenerative Markers for Psychiatric Conditions Publication Number: 20120196300; *In Vitro* Multiparameter Determination Method for the Diagnosis and Early Diagnosis of Neurodegenerative Disorders Publication Number: 20100062463; *In Vitro* Method for the Diagnosis and Early Diagnosis of Neurodegenerative Disorders Publication Number: 20100035286; *In Vitro* Procedure for Diagnosis and Early Diagnosis of Neurodegenerative Diseases Publication Number: 20090263822. *In Vitro* Method for the Diagnosis of Neurodegenerative Diseases Patent Number: 7547553; CSF Diagnostic *In Vitro* Method for Diagnosis of Dementias and Neuroinflammatory Diseases Publication Number: 20080206797; *In Vitro* Method for the Diagnosis of Neurodegenerative Diseases Publication Number: 20080199966; Neurodegenerative Markers for Psychiatric Conditions Publication Number: 20080131921. SV is an officer and director of NeuroVision. LA-A is an employee of NeuroVision. EE is the unique owner of 2E Science, a for-profit private scientific company. Neither EE nor 2E Science have any commercial interest or financial tie in relation with this article. MW is employed by the company TranScrip Partners, Reading, United Kingdom. He is a clinical consultant of Chiesi Farmaceutici. AV is an employee of Eisai Inc. and received lecture honoraria from Roche, MagQu LLC, and Servier. SL received lecture honoraria from Roche and Servier. The remaining authors declare that the research was conducted in the absence of any commercial or financial relationships that could be construed as a potential conflict of interest.

## Footnotes

^1^https://clinicaltrials.gov/ct2/show/NCT01230853 (A Randomized, Double-blind, Placebo-controlled, Combined Single Ascending Dose and Multiple Ascending Dose Study to Assess Safety, Tolerability, Immunogenicity, Pharmacodynamic Response, and Pharmacokinetics of Intravenous Infusions of BAN2401 in Subjects With Mild to Moderate Alzheimer's Disease).

## Abbreviations

[^11^C]-PIB: [^11^C]-Pittsburgh compound-B
[^18^F]-fluorodeoxyglucose-PET: [^18^F]-fluorodeoxyglucose-positron emission tomography
Aβ: amyloid beta
Aβ_1−40_: 40-amino acid-long amyloid beta peptide
Aβ_1−42_: 42-amino acid-long amyloid beta peptide
AD: Alzheimer's disease
ADAPT: Alzheimer's disease anti-inflammatory prevention trial
ADCS: Alzheimer's disease cooperative studies
APMI: Alzheimer precision medicine initiative
APMI-CP: Alzheimer precision medicine initiative cohort program
APS: Alzheimer progression score
ASC: apoptosis-associated speck-like protein containing a caspase recruitment domain
BDNF: brain-derived neurotrophic factor
CB2R: cannabinoid receptor type 2
CCE: cell cycle events
CD: cell surface cluster of differentiation
CDx: companion diagnostic
CNS: central nervous system
COX: cyclooxygenase
COX-1: cyclooxygenase-1
COX-2: cyclooxygenase-2
CRP: C-reactive protein
CSF: cerebrospinal fluid
CSF-1R: colony stimulating factor 1 receptor
GWAS: genome-wide association studies
ILs: interleukins
IL-1Ra: interleukin-1 receptor antagonist
INTREPAD: impact of naproxen treatment in presymptomatic Alzheimer's disease
JNK: c-Jun Kinase
K-ARPI: Korean AD research platform initiative based on immune-inflammatory biomarkers
LTP: long-term potentiation
MAPK: p38 mitogen-activated protein kinase
MCI: mild cognitive impairment
MCP-1: monocyte chemoattractant protein-1
MMP-9: metalloprotease-9
MMSE: Mini-Mental State Examination
mNGF: mature nerve growth factor
Nap1: Nck-associated protein 1
ND: neurodegenerative diseases
NF-κB: nuclear factor kappa-light-chain-enhancer of activated B cells
NFT: neurofibrillary tangles
NGF: nerve growth factor
NIH: National Institutes of Health
NSAIDs: non-steroidal anti-inflammatory drugs
P4M: predictive, preventive, personalized and participatory medicine
PET: positron emission tomography
PMI: US precision medicine initiative
PMI-CP: US PMI cohort program
proNGF: precursor of the nerve growth factor
p-tau: hyperphosphorylated tau
RNS: reactive nitrogen species
ROS: reactive oxygen species
SCARA1: class A1 scavenger receptor
SphK1: sphingosine kinase 1
sTREM2: soluble TREM2
TGF-β1: transforming growth factor-beta1
TLRs: Toll-like receptors
TNF-α: tumor necrosis factor-alpha
TNF-Rs: TNF receptors
TNF-RI: TNF receptor I
TNF-RII: TNF receptor II
TREM2: triggering receptor expressed on myeloid cells 2
TSPO: translocator protein.
